# Revisiting psychiatry’s relationship with spirituality

**DOI:** 10.3389/fpsyt.2024.1441922

**Published:** 2024-07-18

**Authors:** Katrina DeBonis

**Affiliations:** ^1^ Department of Psychiatry and Biobehavioral Sciences, David Geffen School of Medicine, University of California, Los Angeles, Los Angeles, CA, United States; ^2^ US Department of Veterans Affairs Greater Los Angeles Healthcare System, Los Angeles, CA, United States

**Keywords:** psychopathology, spirituality, psychedelic-assisted therapy, psilocybin, MDMA

Over the past three decades in the United States, scholars have observed an alarming rise in “deaths of despair” – a term capturing deaths from suicide, drug overdoses, and alcoholism (1). In May 2023, the United States Surgeon General, Dr. Vivek Murthy, released an advisory describing an epidemic of loneliness and isolation that is having devastating effects on the mental and physical health of our society (2). The use of the terms “despair” and “loneliness” to describe driving forces of health outcomes lends evidence to fundamental human needs for connection and meaning - needs that if not met can negatively impact health. Both connection and meaning are dimensions of spirituality, which has been defined as a dynamic and intrinsic aspect of humanity through which persons seek ultimate meaning, purpose, and transcendence and experience relationship to self, family, others, community, society, nature, and the significant or sacred (3). Spiritual concerns emerge commonly in psychiatric clinical practice, as mental illness often inflicts pain that leads to isolation, hopelessness, and suicidal ideation. Patients struggle with existential questions like “why did this happen to me?” and “what’s the point?” Sometimes, their concerns are more directly spiritual in nature: “If there is a God, why would he let anyone suffer like this?”

Psychiatry has adopted a model of evaluation and treatment that largely doesn’t consider spirituality – as a need or as a resource - despite evidence that patients with mental illness often turn to spirituality to cope and that spirituality can have both negative and positive impacts on people with mental illness (4). Recently, there has been a growing awareness of the connection between spirituality and health outcomes. In 2016, The World Psychiatric Association published a position statement urging for spirituality and religion to be included in clinical care (5) and a recent review of spirituality and health outcome evidence led to the recommendation that health care professionals recognize and consider the benefits of spiritual community as part of efforts to improve well-being (3). Within the context of public mental health services, spiritual needs have been considered through developing opportunities for people to nurture meaningful connections with themselves, others, nature, or a higher power (6). Recognizing the spiritual needs of patients approaching the end of their life, the field of hospice and palliative medicine, in contrast to psychiatry, explicitly identifies the need for palliative medicine physicians to be able to perform a comprehensive spiritual assessment and provide spiritual support (7).

Psychiatry’s framework leads us to make diagnoses and consider evidence-based treatments such as medications and psychotherapy which are successful for some people, some of the time, and to some degree. Those who do not benefit from these interventions then progress through the best we currently have to offer in our treatment algorithms, often involving multiple attempts at switching and adding medications in combination with psychotherapy, if accessible. Evidence-based medicine in psychiatry relies on efforts to turn subjective experiences into objective metrics that can be measured and studied scientifically. This pursuit is important and necessary to fulfill our promise to the public to provide safe and effective treatment. As doctors and scientists, it is also our responsibility to acknowledge the limits of objectivity when it comes to our minds as well as the illnesses that inhabit them and allow for the subjective and intangible aspects of the human condition to hold value without reduction or minimization of their importance. The limits of our empirical knowledge and the legitimacy of the subjective experience, including mystical experiences, in the growing body of psychedelic research offers psychiatry an opportunity to reconsider its relationship with spirituality and the challenges and comforts it brings to those we seek to help.

In his book, *The Future of an Illusion*, Sigmund Freud wrote “Religion is a system of wishful illusions together with a disavowal of reality” (8) a stance which has likely had far-reaching implications on how psychiatrists regard religion and spirituality, with psychiatrists being the least religious members of the medical profession (9). In his subsequent work, *Civilization and its Discontents*, Freud describes a letter he received from his friend and French poet, Romain Rolland, in which the poet agreed with Freud’s stance on religion but expressed concern with his dismissal of the spiritual experience. Freud wrote of his friend’s description of spirituality:

“This, he says, consists in a peculiar feeling, which he himself is never without, which he finds confirmed by many others, and which he may suppose is present in millions of people. It is a feeling which he would like to call a sensation of ‘eternity,’ a feeling as of something limitless, unbounded—as it were, ‘oceanic’ (10)”.

Almost a hundred years later, the experience of oceanic boundlessness and related experiences of awe, unity with the sacred, connectedness, and ineffability, are now commonly assessed in psychedelic trials through scales such as the Mystical Experiences Questionnaire and Altered States of Consciousness questionnaire. Although an active area of debate, there is evidence that these spiritual or mystical experiences play a large part in mediating the therapeutic benefit of psychedelic treatment (11)​. In a systematic review of 12 psychedelic therapy studies, ten established a significant association between mystical experiences and therapeutic efficacy (12). Although this may not be surprising given that psychedelic compounds have been used in traditional spiritual practices for millennia, these findings from clinical trials provide evidence to support Rolland’s concerns to Freud about the importance of spiritual experiences in mental health.

Later in *Civilization and its Discontents*, Freud admits “I cannot discover this ‘oceanic’ feeling in myself. It is not easy to deal scientifically with feelings… From my own experience I could not convince myself of the primary nature of such a feeling. But this gives me no right to deny that it does in fact occur in other people (10).” We can acknowledge the inherent limits that would underlie the field of psychoanalysis Freud created with his explicit disdain for religion and lack of experiential understanding of the benefits of spiritual experiences. To see patients with mental illnesses that have been labeled treatment resistant experience remarkable benefit from feelings of transcendence catalyzed by psilocybin should lead us with humility to question what unmet needs might underlie treatment resistance and to reexamine the role of spirituality and connectedness in the prevention, evaluation, and treatment of mental illness. Not everyone with mental illness will be a good candidate for treatment with psychedelic medicine, but every individual is deserving of treatment that considers our need and potential sources for connection, meaning, and transcendence.

## Author contributions

KD: Conceptualization, Data curation, Formal analysis, Funding acquisition, Investigation, Methodology, Project administration, Resources, Software, Supervision, Validation, Visualization, Writing – original draft, Writing – review & editing.
